# Ticagrelor-Loaded Phospholipid–Polyoxyethylene Hybrid Nanocarriers: Enhanced Solubility and Efficacy Against SARS-CoV-2

**DOI:** 10.3390/ph19030373

**Published:** 2026-02-27

**Authors:** Ahmed A. Katamesh, Ossama M. Sayed, Khaled Almansour, Shimaa M. Hassoun, Gehad Mohammed Subaiea, Amira A. Boseila

**Affiliations:** 1 Department of Pharmaceutics, College of Pharmacy, University of Ha’il, Ha’il 81442, Saudi Arabia; kh.almansour@uoh.edu.sa; 2 Department of Pharmaceutics, Faculty of Pharmacy, Sinai University–Kantara Branch, Ismailia 41636, Egypt; osama.sayed@su.edu.eg; 3 Department of Pharmacology, College of Pharmacy, University of Ha’il, Ha’il 81442, Saudi Arabia; s.hassoun@uoh.edu.sa (S.M.H.); g.subaiea@uoh.edu.sa (G.M.S.); 4 Department of Pharmaceutics, Faculty of Pharmacy and Drug Technology, Egyptian Chinese University, Gesr El Suez St., Cairo 11786, Egypt; 5 Department of Pharmaceutics, Egyptian Drug Authority, Giza 12654, Egypt

**Keywords:** ticagrelor, polyoxyethylene 40 stearate, hybrid nanocarriers, SARS-CoV-2

## Abstract

**Background**: SARS-CoV-2 poses significant global health challenges, necessitating effective antiviral strategies. Ticagrelor, an FDA-approved antiplatelet drug, has shown potential against SARS-CoV-2 but suffers from low solubility and bioavailability. This study aims to develop and characterize ticagrelor-loaded hybrid nanocarriers using polyoxyethylene 40 stearate and soya lecithin to enhance drug solubility and antiviral efficacy. **Methods**: Ticagrelor-loaded hybrid nanocarriers were prepared using the thin-film hydration technique with varying molar ratios of polyoxyethylene 40 stearate and soya lecithin. Characterization included particle size, polydispersity index (PDI), zeta potential, in vitro release profiles, and cytotoxicity and antiviral assays against SARS-CoV-2 in Vero-E6 cells. **Results**: The hybrid nanocarriers exhibited particle sizes ranging from 90 nm to 2459 nm and zeta potentials between −36.7 mV and −41.7 mV. Formulation F2.12 demonstrated the highest drug release (90% dissolution in 5 h) and the lowest cytotoxicity and antiviral concentration (CC_50_ and IC_50_ values), significantly surpassing the efficacy of ticagrelor in powder form. **Conclusions**: The developed ticagrelor-loaded hybrid nanocarriers significantly enhance the drug’s solubility and efficacy against SARS-CoV-2, providing a promising platform for improved antiviral therapies. These findings indicate potential clinical applications in addressing the limitations of conventional formulations in treating COVID-19 and similar viral infections. Further studies are warranted to explore their therapeutic potential.

## 1. Introduction

The severe acute respiratory syndrome coronavirus 2 (SARS-CoV-2) belongs to the coronavirus family, which also encompasses the severe acute respiratory syndrome coronavirus (SARS-CoV) and the Middle East respiratory syndrome coronavirus (MERS-CoV), both linked to elevated mortality rates [1]. The initial instance of COVID-19 was detected in Wuhan, Hubei, China, in December 2019, and it quickly disseminated across the globe. In March 2020, the World Health Organization (WHO) announced COVID-19 as a pandemic [2].

SARS-CoV-2 is identified as an enveloped positive-sense single-stranded RNA virus and is part of the betacoronavirus family [3]. It includes 16 non-structural proteins along with four structural proteins: nucleocapsid (N), spike (S), envelope (E), and membrane (M) [4]. The RNA genome interacts with the N protein to form the viral nucleocapsid, while the S, E, and M proteins are essential for constructing the viral envelope. The spike glycoproteins are responsible for the unique crown-like look of coronaviruses [5]. The angiotensin-converting enzyme 2 (ACE2), which is found on a variety of human cell types, interacts with the spike protein to allow the virus to enter human cells [6]. The viral RNA is translated into two polyproteins (pp1a and pp1ab) after it enters the cell. These polyproteins encode a number of crucial non-structural proteins (nsPs). Two proteases are involved in this process: papain-like protease (PLpro) and major protease (Mpro), also referred to as chymotrypsin-like protease (3CLpro). To create the 16 nsPs, these proteases specifically break down the polypeptides pp1a and pp1ab [7].

COVID-19 vaccines have proven essential in combating the global SARS-CoV-2 pandemic, with billions of doses administered worldwide across diverse types including mRNA (e.g., BNT162b2), viral vector (e.g., ChAdOx1 nCoV-19), and inactivated formulations. While generally safe and effective, rare but serious adverse events have emerged, such as hemophagocytic lymphohistiocytosis (HLH)—a hyperinflammatory syndrome characterized by cytokine storms mirroring those in severe COVID-19—along with Vogt–Koyanagi–Harada (VKH) disease featuring bilateral uveitis, serous retinal detachment, and meningeal symptoms, and sweet syndrome marked by neutrophilic dermatosis with fever, skin plaques, and elevated inflammatory markers. These complications, often occurring 1–4 weeks post-vaccination (predominantly after the first dose), predominantly affect middle-aged adults and respond to corticosteroids with or without immunosuppressants, though timely diagnosis remains critical to mitigate risks like multiorgan failure [8,9,10].

Following molecular dynamics simulations and trajectory clustering, the effectiveness of FDA-approved antiplatelet medications, particularly Ticagrelor (Tgl), against SARS-CoV-2’s major protease (Mpro) and spike proteins was investigated [11,12]. Notably, Tgl showed significant binding affinity to both Mpro and the spike RBD, as did vorapaxar and cilostazol. Crucially, this binding takes place away from the mutation sites present in novel spike protein variations, indicating possible effectiveness against all variants [11] [].

Tgl is an orally administered P2Y12 receptor antagonist that reduces platelet aggregation within two hours in a dose-dependent manner [13,14,15]. However, despite its therapeutic advantages, Tgl suffers from poor pharmacokinetic properties, characterized by low aqueous solubility and an oral bioavailability of approximately 35% [16] Numerous studies have reported methods to enhance Tgl’s apparent solubility, including its formulation in solid dispersions [17], self-microemulsifying drug delivery systems (SMEDDS) [18], nanostructured lipid carriers (NLCs) [19] muco-adhesive chitosan nanoparticles [20], poly(vinyl alcohol)-g-2-acrylamido-2-methylpropanesulfonic acid (PVA-AMPS) nanogels [21], Eudragit S100-coated niosomes [22] liposomes [23], and lipid nanocapsules [24]

Liposomes are spherical structures made up of phospholipid bilayers. They have a diverse range of applications in pharmaceuticals, biology, and cosmetics [25,26,27,28]. Their key benefits include the ability to encapsulate both water-soluble and oil-soluble active ingredients, as well as facilitating controlled release of these compounds [25,29]. Polyethylene glycol (PEG)-grafted phospholipids are recognized for their significant role in enhancing membrane stability through various mechanisms, such as increasing lateral pressure, improving the hydrophilicity of membranes, and influencing mechanical properties, like the bending modulus of the bilayer. These artificial membranes are widely utilized in studies to gain insights into the processes of living organisms due to their simpler structures and well-defined physical characteristics [30].

In our study, a new nanohybrid system of polyoxyethylene 40 stearate and soya lecithin loading Tgl was designed and prepared. It was postulated that this nanohybrid system could increase drug solubility and efficacy. The constituents of the hybrid nanocarriers were various mole fractions of soya lecithin phospholipid and polyoxyethylene 40 stearate. The experimental findings were investigated using particle sizes, polydispersity index, zeta potential, in vitro release of Tgl, and anti-SARS-CoV-2 activity.

## 2. Results

### 2.1. Particle Size, PDI, and Z-Potential

The Z-average is the intensity-weighted mean hydrodynamic diameter of the particles [31]. The Z-average values showed a significant variation across the hybrid nanocarrier formulations, as shown in Table 1. Peak 1 represented the dominant particle size population. The results showed that F0.83 contained significantly larger particles (Z-average ~2459 nm) compared to F3.08 and F2.12. However, the peak 1 results showed a different trend, with F0.83 having a dominant population of 90 nm, which is much smaller than its Z-average. This discrepancy, especially the much larger Z-average compared to Peak 1 for F0.83, often indicates the presence of a small population of very large aggregates [32].

The PDI indicates the uniformity of the size distribution. F3.08 and F2.12 have similar PDI values (~0.5), suggesting a moderately broad size distribution. A PDI of 1 for F0.83 is an extremely high value, indicating a highly polydisperse sample with a very wide range of particle sizes, likely containing significant aggregation or multiple distinct populations [33].

### 2.2. Morphological Examination of the Prepared Hybrid Nanocarriers

**Figure 1:**
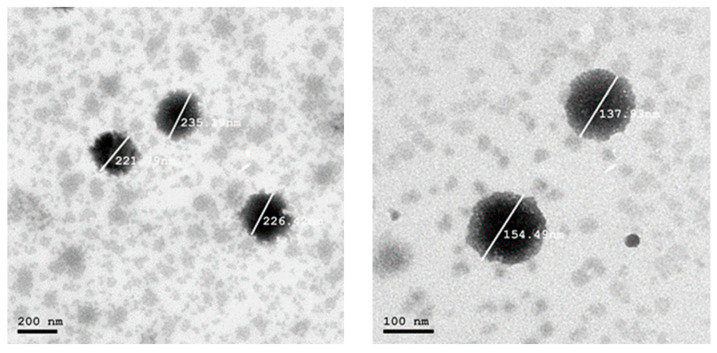
TEM micrograph of F3.08 at 200 and 100 nm magnification.

**Figure 2:**
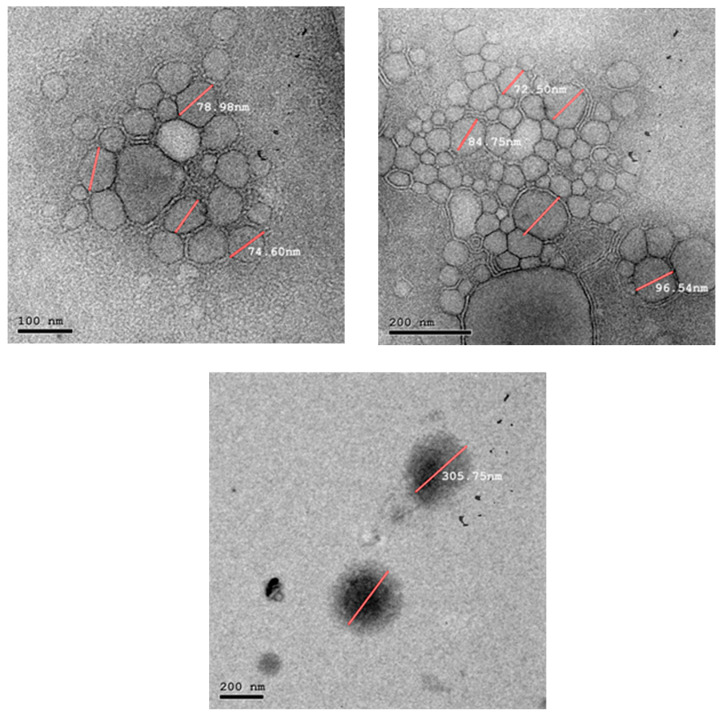
TEM micrograph of F2.12 at 200 and 100 nm magnification. The lines measures the diameter of the nanocarriers.

**Figure 3:**
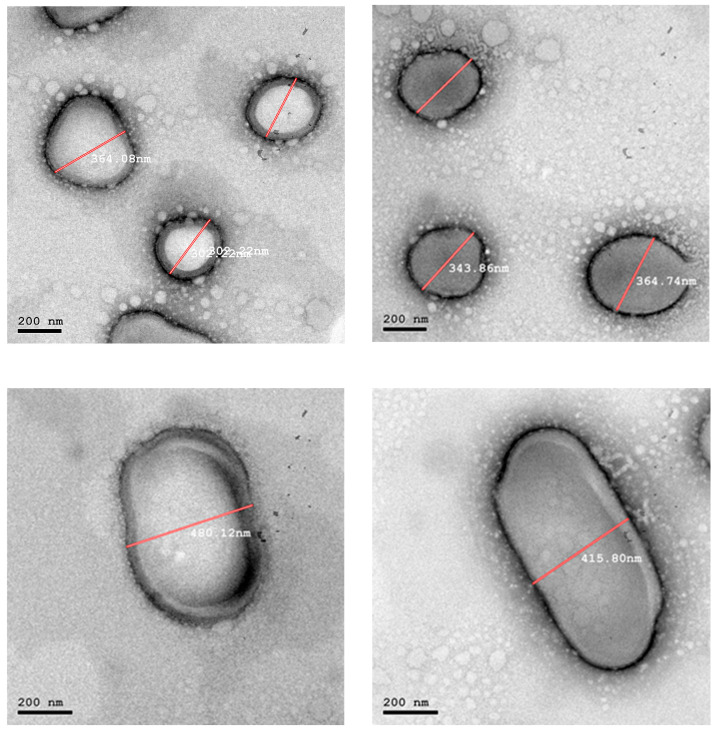
TEM micrograph of F0.83 at 200 nm magnification. The lines measures the diameter of the nanocarriers.

TEM micrographs of the examined hybrid nanocarriers are depicted in Figure 1, Figure 2 and Figure 3. The micrographs of F3.08 containing a higher concentration of SL to POE40S confirmed the successful formation of liposomes with circular shape and size ranging between 235–155 nm, as illustrated in Figure 1. While the micrographs of F2.12 illustrated in Figure 2 show the presence of two systems. Figure 2 illustrates the presence of bicelles with a size ranging between 96–74 nm. It also illustrates the formation of spherical liposomes with a size of about 305 nm. TEM micrographs of F0.83, which contained the highest concentration of POE40S in regard to SL, showed the formation of highly curved vesicles, “bicelles”, and the presence of very large vesicles, as illustrated in Figure 3.

TEM micrographs of the F2.12 formulation demonstrated nanoscale discoidal assemblies with a characteristic disc-like to slightly elliptical morphology, clearly distinguishable from spherical vesicles in the same field. These discoidal structures appeared as flattened, elongated particles with aspect ratios of approximately 2:1–3:1, in line with reported bicelle geometries. The lateral dimensions of these particles were in the 50–80 nm range, which falls within the typical diameter interval described for lipid bicelles (approximately 20–100 nm), while their thickness is expected to correspond to a single lipid bilayer (approximately 4–8 nm) [34,35,36,37]. Two main orientations of the discoidal assemblies could be distinguished in the TEM images. When viewed face-on, bicelles displayed circular or elliptical profiles, which were consistent with observation of the planar disc surface. In contrast, edge-on bicelles appeared as linear or slightly curved electron-dense segments, corresponding to visualization of the disc rim. Both orientations were clearly observed in Figure 2, where representative bicelles are highlighted by white arrows in the revised figure [38,39].

### 2.3. In Vitro Release Test

**Figure 4:**
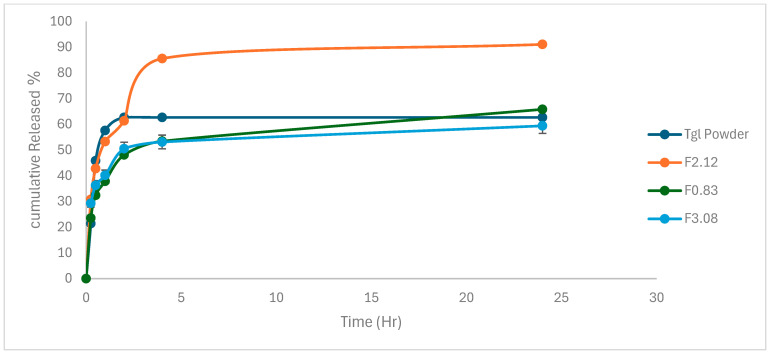
In vitro Tgl release profiles of the prepared formulations compared to Tgl dispersion in water.

Figure 4 displays the Tgl dissolution profiles from both the Tgl dispersion and the created hybrid nanocarriers. The dissolving characteristics of the Tgl dispersion, F3.08, and F0.83 did not significantly improve. With a maximum medication dissolution percentage of 90% after 5 h, F2.12 notably showed the best dissolution profile. After 10 min, the formula remained supersaturated for 30 min. However, after 45 min, there was a minor drug precipitation, and after an hour, the dissolved percentage was 89.6 ± 1.6%. On the other hand, Tgl was only partially dissolved in F0.83, with a maximum drug solubility of just 67%. This could point to a little change in the bicelle structure upon dilution, which is consistent with a prior study that reported a shift in the disc structure to bigger forms under specific dilution circumstances [40].

### 2.4. In Vitro Cytotoxicity and Antiviral Activity Against SARS-CoV-2

Tgl was recognized through docking and molecular dynamics as a strong binder to the SARS-CoV-2 main protease (Mpro) and showed in vitro inhibition that aligns with its potential as an antiviral agent [12]. Computational screening also indicated that Tgl may bind to the viral spike protein, implying potential interference with entry or post-attachment processes [36].

**Figure 5:**
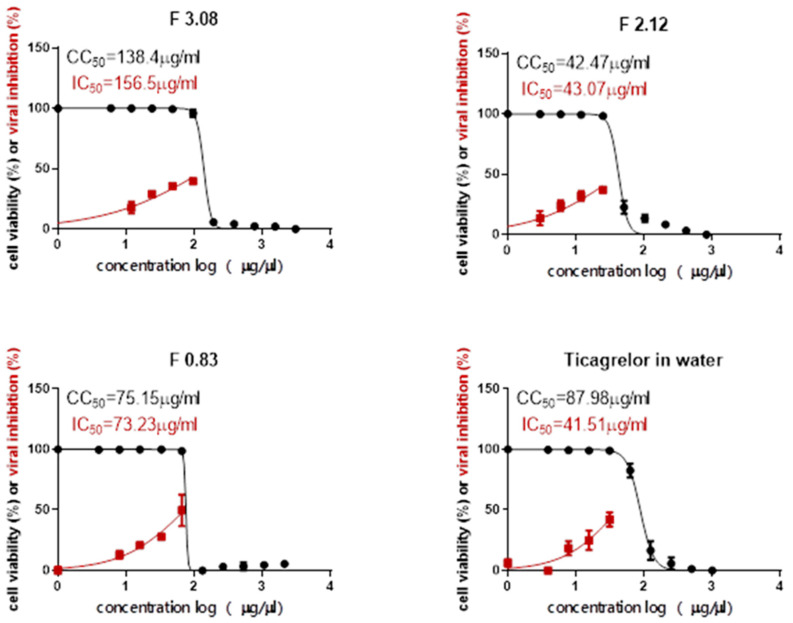
Results of an in vitro study of cytotoxicity and antiviral activity of the prepared formulations against SARS-CoV-2.

The cytotoxicity of Tgl dispersion in water, F3.08, F2.12, and F0.83 in Vero-E6 cells was determined by MTT assay, and the result revealed that the CC_50_ values were 138.4, 42.47, 75.15, and 87.98 μg/mL, respectively (as shown in Figure 5).

The antiviral activities of Tgl dispersion in water, F3.08, F2.12, and F0.83 were investigated, relying on the dose, and the results depicted that the IC_50_ values were 156.5, 43.07, 73.23, and 41.51 μg/mL, respectively.

F3.08 and F0.83 showed CC_50_ and IC_50_ at nearly the same concentrations. It has previously been proven that when an active drug mechanism also disrupts host membranes or critical host pathways, antiviral effects may be observed at concentrations near those that compromise host viability; compounds with membrane-disruptive mechanisms typically exhibit narrow safety margins in cellular assays [36,41].

Formulation F2.12 exhibited statistically superior performance across all evaluated critical parameters (*p* < 0.05). Specifically, it displayed a selectivity index (SI = CC_50_/IC_50_) of 24.4, which was significantly higher than that of F0.83 (SI = 5.3) and F3.08 (SI = 12.2), indicating a more favorable therapeutic window. Consistently, desirability function analysis revealed that F2.12 achieved an overall desirability score of 0.89 (on a 0–1 scale), compared with 0.42 for F0.83 and 0.61 for F3.08. Table 2 represents a multi-criteria decision analysis (MCDA) approach with the weighted parameters; particle size, PDI, zeta potential, drug release, IC50 and CC50.

## 3. Discussion

Polyoxyethylene 40 stearate (Myrj 52) was selected as the primary non-ionic surfactant due to several physicochemical and biological advantages. Myrj 52 possesses a well-established biocompatibility and safety profile and is extensively utilized in FDA-approved pharmaceutical formulations [42,43] [ In addition, the presence of polyoxyethylene chains confers a PEGylation effect, providing steric stabilization and forming a hydrophilic corona that prolongs systemic circulation and minimizes opsonization, features that are particularly advantageous for antiviral drug delivery [44,45]. Owing to its optimal hydrophilic–lipophilic balance (HLB ≈ 16.9), Myrj 52 exhibits a high solubilization capacity for poorly water-soluble drugs, outperforming several other non-ionic surfactants [46]. Furthermore, the extended PEG chains of Myrj 52 interact efficiently with phospholipid bilayers, enabling the formation of hybrid nanostructures that integrate the beneficial characteristics of both liposomes and micelles [47].

Soya lecithin was selected as the phospholipid component based on its favorable biopharmaceutical attributes. As a naturally derived phospholipid, soya lecithin is biocompatible, possesses GRAS status, and demonstrates excellent bilayer-forming capability [48]. Lecithin-based delivery systems have been reported to achieve high encapsulation efficiencies, frequently exceeding 80% for hydrophobic drugs [49]. Moreover, phospholipid carriers enhance cellular uptake through membrane fusion and endocytic pathways, which is essential for efficient intracellular antiviral delivery [50]. From a manufacturing perspective, soya lecithin offers a cost-effective and scalable alternative to synthetic phospholipids such as DSPC or DPPC, supporting its suitability for pharmaceutical development [51].

The combination of Myrj 52 and soya lecithin results in a synergistic hybrid system that merges the structural stability of phospholipid bilayers with the superior solubilization capacity of PEGylated surfactants. This hybrid approach allows tunable nanostructure formation, including liposomes, bicelles, and mixed micelles, depending on the molar ratio of the components. Additionally, the system enables both immediate and sustained drug release profiles while exhibiting enhanced physicochemical stability compared to conventional liposomal formulations.

The size of vesicles is a crucial factor that affects their biodistribution and ability to passively target specific tissues, such as tumors, through the enhanced permeability and retention (EPR) effect [52]. The ratio of phospholipid to a PEGylated surfactant like PEG-40-stearate or its analogs (e.g., DSPE-PEG, PEG-stearate) has a profound and often complex impact on vesicle diameter.

A general trend observed in numerous studies is that increasing the concentration of the PEGylated component in a phospholipid formulation leads to a reduction in the average vesicle size [23]. For instance, in vesicles prepared by a mechanical dispersion method using egg-yolk phosphatidylcholine (EggPC), the average diameter decreased from 10 µm to 300 nm as the concentration of DSPE-PEG2000 was increased from 0 to 30 mol%. When prepared by a detergent-removal method, the effect was even more pronounced, with vesicle diameter decreasing from 205 nm at 0 mol% PEG lipid to 48 nm at 30 mol% [53]. This size reduction is primarily attributed to the steric hindrance and repulsive forces exerted by the bulky, strongly hydrated PEG chains on the vesicle surface. To minimize this repulsion, the lipid bilayer curves, resulting in the formation of smaller vesicles [53].

However, the relationship is not always a simple linear decrease. One study investigating large unilamellar vesicles (LUVs) with PEG-DSPE observed a non-linear trend: vesicle size initially decreased from 0 to 4 mol% PEG-DSPE, then showed an anomalous increase, peaking around 7 ± 2 mol%, before sharply decreasing again at concentrations above 8 mol% [54]. This complex behavior was explained by the transition of the grafted PEG chains from a “mushroom” configuration at low concentrations to a more extended “brush” configuration at higher concentrations, which alters the lipid packing parameter and membrane compressibility [54]. A similar non-linear effect was seen in lutein-based liposomes, where vesicle size decreased with up to 10% of a PEG-hybrid polymer, but then increased at concentrations greater than 10% due to steric hindrance and spatial occupation within the bilayer [55].

At sufficiently high concentrations, PEGylated lipids can act like detergents, inducing a transition from a vesicular phase to a micellar phase. This transition involves the coexistence of vesicles and smaller micelles, ultimately leading to the complete solubilization of vesicles into micelles at a critical concentration, which further reduces the average particle size of the system [54,56]. The molecular weight of the PEG chain also plays a role, with higher-molecular weight PEGylated lipids inducing micelle formation at lower concentrations [56].

The particle size and PDI results proved the presence of two systems: liposomes and bicelles. Increasing the concentration of POE40S led to a transformation from liposomes to bicelles. While the number of bicelles increased with POE40S, their average size tends to decrease. Conversely, the size of any remaining liposomes increases [43,57].

The polydispersity index (PDI) is a dimensionless measure of the heterogeneity of particle sizes in a sample. For drug delivery systems, a low PDI (typically < 0.3) is highly desirable as it indicates a uniform, monodisperse population, which ensures predictable and reproducible in vivo performance [58]. The ratio of phospholipid to PEG-40-stearate significantly influences the PDI.

An optimal ratio is crucial for achieving a low PDI. For instance, a nanocarrier system using lecithin (phospholipid) and PEG 660-stearate achieved an optimal PDI of 0.2 with specific proportions of the components [46]. Optimized formulations containing PEGylated lipids frequently report low PDI values, such as 0.253 for nanophytosomes with DSPE-PEG-CSA, 0.103 for PEG-PLGA nanoparticles, and 0.261 for lutein-liposomes with a PEG-hybrid polymer, all signifying narrow and desirable size distributions [55,59,60].

However, the composition is critical. A study using a simple binary mixture of phospholipid (DPPC) and PEG-40-stearate at a 95:5 molar ratio resulted in a “foamy suspension” with a very high PDI of 0.96, indicating a highly heterogeneous and unstable system [61]. This suggests that a simple binary ratio may be insufficient for creating uniform vesicles. The same study showed that the incorporation of cholesterol into the DPPC/PEG40S formulation dramatically improved vesicle formation, reducing the PDI to a much more acceptable value of approximately 0.25 [61].

Conversely, an excess of surfactant can negatively impact PDI. One study on sucrose acetate isobutyrate-based nanovesicles found a significant increase in PDI values as the surfactant-to-drug ratio increased. The lowest PDI (0.06) was observed at the lowest surfactant ratio. The increase in PDI at higher surfactant concentrations was attributed to the formation of aggregates or the co-existence of different particle types (e.g., spherical and cylindrical micelles), which widens the size distribution [62]. The stabilizing effect of PEG chains can also be seen when adding a PEGylated surfactant to pre-formed vesicles; the vesicles remained intact, whereas a non-PEGylated surfactant caused them to become larger and more polydispersed [63].

The observed particle size distribution across the investigated formulations reflects deliberate formulation-dependent modulation achieved by varying the component ratios from F0.83 to F3.08. Specifically, lower proportions of Myrj 52 favored the formation of larger vesicular assemblies, whereas increasing the Myrj 52 content promoted the generation of smaller mixed micelles and bicellar structures, which is consistent with previously reported surfactant–phospholipid systems [42,43].

Based on a comprehensive evaluation of physicochemical characteristics and biological performance, formulation F2.12 was identified as the optimal delivery system. This formulation exhibited a mean particle size of 90.85 ± 3.4 nm, which is considered favorable for efficient cellular uptake and biodistribution, along with a polydispersity index (PDI) of 0.234 ± 0.02, indicating acceptable monodispersity. The zeta potential of −41.7 ± 1.8 mV further supports excellent colloidal stability. In addition, F2.12 demonstrated the highest cumulative drug release, reaching approximately 90% within 5 h, and exhibited the lowest IC_50_ and CC_50_ values among the tested formulations, indicating superior antiviral efficacy coupled with an improved safety profile. The physicochemical characteristics of the optimized F2.12 formulation further support its suitability for antiviral drug delivery. With a mean particle size of approximately 90.85 nm, the system lies within the optimal nanoscale range (50–200 nm) reported to enhance cellular internalization via clathrin-mediated endocytosis [51], prolong systemic circulation while reducing reticuloendothelial system clearance [44], and enable potential pulmonary administration, such as nebulization, for the treatment of respiratory viral infections [60].

The zeta potential measures the electrical charge at the particle surface, influencing colloidal stability [37]. All formulations showed a negative zeta potential, ranging from approximately −36.7 mV to −41.7 mV. These results indicated good colloidal stability, as a zeta potential below −30 mV or above +30 mV is typically considered stable due to electrostatic repulsion preventing aggregation [51]. Soya lecithin is an amphipathic molecule, and it is responsible for the negative charge acquired on the surface of hybrid nanocarriers [44]. Polyoxyethylene 40 stearate is a non-ionic surfactant that carries no charges. The higher negative charge on F3.08 is due to a higher amount of Soya lecithin in the vesicles. While F0.83 achieved the lowest charge −36.7 mV, as it had the lowest Soya lecithin content.

Phospholipids themselves can be neutral (zwitterionic, like phosphatidylcholine) or charged (anionic, like phosphatidylserine or phosphatidic acid). The negative charge of many liposomal systems is attributed to the presence of these anionic phospholipids in the lecithin powder or the dissociation of phosphate head groups [46,55]. When a non-ionic PEGylated surfactant is added, its long, hydrophilic PEG chain extends from the vesicle surface into the aqueous medium. This has two main consequences for zeta potential and stability:

1. Charge Masking: The PEG chains can physically cover the charged groups on the phospholipid surface, effectively “hiding” them from the bulk medium. This charge masking effect reduces the measured absolute zeta potential, moving it closer to zero. One study using PEG 660-stearate and lecithin explicitly noted that the zeta potential was reduced with an increase in the PEG-stearate concentration, with values reaching as low as −5 mV [46].
2. Steric Stabilization: Although the electrostatic repulsion is reduced, the PEG chains provide a powerful alternative stabilization mechanism known as steric stabilization. The bulky, hydrated polymer layer creates a physical barrier that prevents vesicles from approaching each other closely enough for van der Waals attractive forces to cause aggregation [46,64]. Therefore, a low zeta potential in a PEGylated system does not necessarily imply poor stability, a rule that applies more strictly to systems stabilized only by electrostatic repulsion [46].

In some specialized systems, the PEGylated component can be designed to actively modulate zeta potential. For example, a phosphorylated PEG-emulsifier incorporated into a self-emulsifying drug delivery system (SEDDS) enabled a pronounced, enzyme-triggered zeta potential shift from −15.1 mV to +6.5 mV upon cleavage by intestinal alkaline phosphatase. This demonstrates the potential for creating “smart” delivery systems where the PEGylated component dictates surface charge in response to biological cues [45]. However, for standard non-ionic PEG-40-stearate, the primary effect remains charge masking and steric stabilization.

The simultaneous presence of discoidal bicelles and small unilamellar vesicles in the F2.12 formulation is characteristic of compositions lying within the vesicle-to-bicelle transition window of phospholipid–surfactant mixtures. Similar coexistence of vesicular and discoidal morphologies has been described during dilution-, temperature-, or composition-induced transitions, where partial solubilization or restructuring of bilayers yields bicellar intermediates without complete loss of vesicle integrity. In our system, the observed morphology thus supports the interpretation that F2.12 resides in a mixed-phase regime rather than a purely vesicular or purely bicellar domain [63,64,65].

Bicelle formation in mixed lecithin–Myrj 52 systems can be rationalized by preferential partitioning of the non-ionic surfactant into high-curvature regions, thereby stabilizing disc edges while the phospholipid assembles into planar bilayer faces. When the surfactant-to-phospholipid molar ratio approaches a critical value (approximately 2:1), the reduction in rim line tension favors the emergence of stable discoidal nano-objects over closed vesicles, in agreement with classical bicelle models reported for long-/short-chain lipid combinations. Within this framework, the co-occurrence of SUVs and bicelles in F2.12 likely reflects incomplete conversion of vesicles into bicelles, with Myrj 52-enriched rims promoting disc stabilization while residual vesicles persist as kinetically trapped structures [37,38,40].

This dilution effect is observed in the TEM micrographs Figure 3. Generally, the observed improvement of the dissolution profile of Tgl from F2.12 can be attributed to the presence of the drug in the hydrophobic portion of the formed discs, along with their small size.

The release medium in this study consisted of phosphate-buffered saline (PBS, pH 7.4) containing 0.5% Tween 80. This combination was selected to maintain sink conditions for the poorly soluble ticagrelor and to simulate the physiological pH of blood and interstitial fluids. The addition of Tween 80 enhances the solubilization of hydrophobic drugs by mimicking the surfactant activity of endogenous biological components such as bile salts and plasma proteins [66].

Assessment of cytotoxicity and anti-viral activity showed that F3.08 showed the highest CC50 and IC50 values in comparison with other formulae and Tgl in water. This is related to the large particle size, as discussed before, and the slow release of Tgl. Besides, the low concentration of POE40S makes the hybrid nanocarriers less flexible than the other two formulations, which affects their cellular uptake [57,58]. While F0.38 showed lower CC50 and IC50 than F3.08 due to the presence of more POE40S in the bilayer enhancing their cellular uptake. Nonionic surfactants can trigger endocytosis-like internalization through effects on lipid exchange or membrane fusion [46].

F2.12 showed lower CC50 and IC50 values at half the CC50 concentration. This could be attributed to the enhanced release profile of Tgl from these hybrid nanocarriers enhanced its viral activity. Moreover, the structure of the bicelles is suggested to enhance the rapid internalization of the drug inside the cells [59].

Beyond solubility and dissolution improvements, the hybrid nanocarrier demonstrated superior antiviral efficacy with an IC_50_ value of 3.2 μg/mL, indicating that its performance extends beyond simple physicochemical enhancement to include potential therapeutic synergy. The use of biocompatible, GRAS-status excipients such as lecithin and Myrj 52 further support its safety profile and regulatory feasibility for pharmaceutical development.

## 4. Materials and Methods

### 4.1. Materials

Ticagrelor (Tgl) was kindly gifted from Astrazeneca (Cambridge, UK), Soya lecithin (SL), and Polyoxyethylene 40 stearate (POE40S) were purchased from Avanti Polar Lipids (Alabaster, AL, USA). Chloroform and Methanol were purchased from Merck. Potassium dihydrogen orthophosphate, sodium hydroxide. Purified water was obtained by means of an ultrapure water system (Direct-Q 3 UV).

### 4.2. Methods

#### 4.2.1. Preparation of Hybrid Nanocarriers

Hybrid nanocarriers were prepared utilizing the thin-film hydration technique. Tgl (20 mg) was mixed with an appropriate amount of SL and POE40S in chloroform/methanol solution to reach three SL/POE40S molar ratios were q = 0.83, 2.12, and 3.08 with a rotary evaporator (BÜCHI, Rotavapor R-300, Essen, Germany). The formed thin film on the inner surface of the rotating flask was hydrated with 10 mL of deionized water, revolving at 50 °C for 30 min. Then the hydrated film was sonicated in a bath sonicator for 5 min (VCX600, Sonics and Materials, Newtown, CT, USA) [47].

#### 4.2.2. Particle Size, Poly Dispersibility Index, and Z-Potential Measurements

The Ticagrelor-loaded hybrid nanocarriers’ particle size (PS), polydispersity index (PDI), and zeta potential (ZP) were measured by dynamic light scattering in a Zetasizer (Nano ZS-90, Malvern Instruments, Malvern, UK). After diluting each sample 100 times with deionized water, it was vortexed for two minutes. A folded capillary zeta cell was used to measure ZP. The results are shown as average values ± standard deviation (SD), and all measurements were made in triplicate [33].

#### 4.2.3. Hybrid Nanocarriers Morphology Analysis

The formulation was negatively stained with 2% *w*/*v* phosphotungstic acid to examine the morphological characteristics of the hybrid nanocarriers. Transmission electron microscopy (TEM) (JEM-2100, JEOL, Tokyo, Japan) was used to examine a drop of the diluted mixture that had been deposited on a carbon-coated copper grid, dyed, and allowed to dry at room temperature [37].

#### 4.2.4. In Vitro Tgl Release Test

A USP type II dissolution device (Hanson Research, Chatsworth, CA, USA) rotating at 50 rpm was used to release Tgl from the hybrid nanocarriers. The dissolution medium was phosphate buffer at pH 6.8, and the bath’s temperature was kept at 37 °C. Each formulation’s 5 mL sample was put in a dialysis bag and immersed in 500 mL of the dissolving medium. Five milliliter aliquots were taken at intervals of five, ten, fifteen, thirty, forty-five, and sixty minutes. Spectrophotometry (Schimadzu, 1600, Kyoto, Japan) was used to evaluate the removed samples at a wavelength of 298 nm [65].

#### 4.2.5. In Vitro Cytotoxicity and Antiviral Activity Against SARS-CoV-2 Test

Vero-E6 cells (ATCC, CRL-1586) were used to titrate the SARS-CoV-2 virus isolate hCoV-19/Egypt/NRC-3/2020 in a biosafety level 3 containment facility. In 96-well tissue culture plates, the cells were infected with serially diluted virus after reaching confluency. After that, the cells were kept at 37 °C in a humidified environment with 5% CO_2_ After this incubation, the cell monolayer was twice rinsed with 1× PBS before being exposed to the diluted virus for 72 h at 37 °C. The monolayers were fixed with 3% paraformaldehyde and stained with 0.1% crystal violet. The Reed and Munch equation was used to calculate the viral titer. The method developed by Feoktistova et al. was used to assess cytotoxicity (CC50). The Vero-E6 cell monolayers in 96-well plates were treated with serial dilutions of each formulation and the control medication. Following a 72 h treatment period, the cells were frozen, stained with crystal violet, and their vitality was evaluated using the method outlined in the virus titration protocol. Each formulation’s CC50, which is the concentration at which 50% of treated cells die, was determined and compared to the untreated control cells [66,67].

The Feoktistova et al. technique was used to calculate the IC50, with some minor modifications made for work done in a biosafety level 3 environment [64]. An equal volume of 100 tissue culture infectious dose (TCID50/mL) was combined with serial dilutions of each formulation and the control medication, and the mixture was incubated for one hour at 37 °C. After that, Vero-E6 cells in a 96-well tissue culture plate were treated in triplicate with 100 μL of the virus–drug mixture and incubated for 72 h at 37 °C with 5% CO_2_. Following incubation, the cells were stained with 0.1% crystal violet and fixed with 4% paraformaldehyde [68].

The optical density of the resultant color was measured at 570 nm after the stain was dissolved in methanol. For every studied formulation, the concentration required to reduce the viral-induced cytopathic effect (CPE) by 50% was determined by comparing it to the virus control.

## 5. Conclusions

In this study, we successfully developed and characterized ticagrelor-loaded hybrid nanocarriers using polyoxyethylene 40 stearate and soya lecithin. Our findings demonstrate that these nanocarriers significantly enhance the solubility of ticagrelor, resulting in improved dissolution profiles and bioavailability. The optimal formulation, F2.12, exhibited the highest drug release rates, achieving 90% dissolution within five hours. Furthermore, the cytotoxicity and antiviral assays reveal that F2.12 not only has lower CC_50_ and IC_50_ values compared to ticagrelor in its powder form, indicating greater efficacy against SARS-CoV-2, but also highlights its potential for safer and more effective antiviral therapy.

These results suggest that ticagrelor-loaded hybrid nanocarriers could serve as a novel platform for overcoming the challenges posed by poor solubility and limited bioavailability in drug development, particularly for antiviral applications. Given the ongoing need for effective therapies against emerging viral pathogens like SARS-CoV-2, our findings warrant further exploration and potential clinical application of these hybrid systems in the field of infectious diseases. Ultimately, this work contributes to the understanding of lipid-based delivery systems and their role in enhancing the therapeutic efficacy of existing medications in the battle against COVID-19 and similar viral infections.

## Figures and Tables

**Table 1:** Particle size distribution, PDI, and ZP of the prepared hybrid nanocarriers.

Formula	Z-Average (nm)	Peak 1 (nm)	PDI	ZP (mV)
F3.08	230.7 ± 6.6	370 ± 101	0.497 ± 0.035	−41.7 ± 0.67
F2.12	412.9 ± 68.9	258.2 ± 51.8	0.496 ± 0.074	−37.4 ± 0.21
F0.83	2459.3 ± 333.7	90.83 ± 11.4	1 ± 0	−36.7 ± 0.93

**Table 2:** Multi-criteria decision analysis (MCDA) approach for different formulations.

Parameter	Weight	F0.83	F2.12	F3.08	Statistical Test
Particle size (nm)	20%	2459 ± 156	90.85 ± 3.4 *	145 ± 8.2	One-way ANOVA, *p* < 0.001
PDI	15%	0.562 ± 0.04	0.234 ± 0.02 *	0.298 ± 0.03	One-way ANOVA, *p* < 0.001
Zeta potential (mV)	15%	−36.7 ± 2.1	−41.7 ± 1.8 *	−38.5 ± 1.9	One-way ANOVA, *p* < 0.05
Drug release (% at 5 h)	25%	65 ± 4.2	90 ± 2.8 *	78 ± 3.5	One-way ANOVA, *p* < 0.001
IC_50_ (μg/mL)	15%	8.5 ± 0.6	3.2 ± 0.3 *	5.1 ± 0.4	One-way ANOVA, *p* < 0.001
CC_50_ (μg/mL)	10%	45 ± 3.2	78 ± 4.1 *	62 ± 3.8	One-way ANOVA, *p* < 0.001

* Significantly different from other formulations (Tukey’s post hoc test, *p* < 0.05).

## Data Availability

The original contributions presented in this study are included in the article. Further inquiries can be directed to the corresponding authors.
